# HIV prevalence, engagement in care, and risk behavior among trans women, San Francisco: Evidence of recent successes and remaining challenges

**DOI:** 10.1177/09564624221111278

**Published:** 2022-07-11

**Authors:** Izzy Chiu, Matisse Leathers, Damiana Cano, Caitlin M Turner, Dillon Trujillo, Sofia Sicro, Sean Arayasirikul, Kelly D Taylor, Erin C Wilson, Willi McFarland

**Affiliations:** 1 1438 University of California Berkeley, Berkeley, CA, USA; 2 Center for Public Health Research, 7152 San Francisco Department of Public Health, San Francisco, CA, USA; 3 Department of Epidemiology and Biostatistics, 8785 University of California, San Francisco, CA, USA

**Keywords:** transgender persons, HIV infections, sexual partners, HIV care, HIV prevention

## Abstract

**Background:**

Trans women have high HIV prevalence and lag behind 90–90-90 targets for HIV care. In San Francisco in 2017, 96% of trans women were aware of their status, 75% were on antiretroviral therapy, 88% had viral suppression. Initiatives to address gaps include peer navigators, free gender-affirming surgery, and housing. Our study updates HIV prevalence and engagement in care among trans women.

**Methods:**

Cross-sectional community-based survey of trans women living in San Francisco sampled by respondent-driven sampling, 7/2019–2/2020 (*N* = 201). Eligibility was: self-identified trans women or other gender and assigned male at birth; living in San Francisco; English/Spanish-speaking; and 18 years or older.

**Results:**

HIV prevalence was 42.3% (95%CI 35.4.-49.4) and associated with having a partner who injected drugs (adjusted odds ratio [AOR] 3.30, 95%CI 1.58–6.90), ever injected drugs (AOR 2.28, 95%CI 1.06–4.89), cost not a barrier to healthcare (AOR 2.63, 95%CI 1.02–6.67), emotional support from family (AOR 2.85, 95%CI 1.43–5.65), and Black/African-American (AOR 2.59, 95%CI 1.16–5.79). Of trans women with HIV, 92.9% were previously diagnosed, 89.9% were on ART, 91.5% reported viral suppression.

**Conclusions:**

Trans women met 90–90–90 targets in 2020, at 93–90–92**.** Interventions need to reach Black/African-American trans women, trans women who inject drugs, and partners of trans women.

## Introduction

Trans women have historically been underrepresented in HIV research despite having the highest prevalence of any population worldwide.^1^ Trans women face barriers to medical care, both gender-related and general care, resulting from gender-related stigma, discrimination, and other structural barriers, with competing unmet basic survival needs.^2^ These barriers are linked to low engagement in HIV care and prevention compared to other populations at risk for or living with HIV in San Francisco.^3,4^ As recently as 2017, the prevalence of HIV among trans women in San Francisco was estimated to be 32.1%, higher than any other population in that city.^5^

Previously identified correlates for HIV prevalence among trans women in San Francisco include African American race/ethnicity, injection drug use, housing instability, and race and gender discrimination.^4,6^ Pre-exposure prophylaxis (PrEP) awareness and use among trans women, while lower than among men who have sex with men (MSM), has increased in San Francisco.^4,7^ PrEP awareness among HIV negative trans women was 79% in early 2018 and 94% in early 2020.^6,7^

An important mechanism to assess the progression of care and treatment for HIV infection is the HIV care continuum or cascade, a simple framework that illustrates access to and retention in care within different stages.^8,9,10^ The HIV care cascade aims to determine priority endpoints for interventions to reach, engage and retain trans women in HIV testing and treatment. The first step of the HIV care cascade measured for trans women in San Francisco in 2017 was 96% aware of their HIV status.^6^ San Francisco Department of Public Health surveillance data for 2017 indicate that 79% of trans women diagnosed with HIV were in care or on antiretroviral therapy (ART), of whom 82% were virally suppressed.^11^

There is great need to be vigilant with the current status of the epidemic response for trans women given the disproportionate burden of HIV, historical gap in access to HIV prevention and care services, and a considerable investment in reducing these disparities in San Francisco. Risk factors for HIV infection help identify points of prevention intervention; gaps in the HIV care cascade guide programs for linkage and retention. Additionally, there are unanswered questions on the epidemiology of HIV among trans women. For example, the source of the extraordinarily high prevalence and persistent new cases of HIV among trans women is uncertain.^12,13,14^ In particular, studies on the partners of trans women are scarce. A few studies suggest the risk may originate with main partners, rather than casual or commercial partners.^15,16^ However, the risk behaviors of partners themselves are largely unknown. Therefore, frequent updates on the current status of the HIV epidemic among trans women are critical to the success in getting to zero HIV infections by 2025.^17^

## Methods

### Design and setting

This is a secondary analysis of cross-sectional survey data collected for the San Francisco site of the National HIV Behavioral Surveillance for Transgender Women (NHBST). Details of the parent survey have been provided by the Centers for Disease Control and Prevention (CDC)^18^ and for a previous analysis of PrEP use among HIV-negative participants.^7,19^ The following briefly describes the key methods and procedures.

### Participants

Participants were recruited from July 2019 to February 2020 by using respondent-driven sampling (RDS) following procedures identical to previous surveys of trans women in San Francisco.^3,5,6,20^ Twenty-five trans women purposively selected from diverse social networks were enlisted as “seeds” to recruit three to five of their peers to the study. Eligibility criteria were self-identified trans women (i.e. currently a woman, trans woman, or other gender and had been assigned male at birth) living in the San Francisco metropolitan statistical area, fluent in English or Spanish, and age 18 years or older. Eligible referrals were in turn asked to refer peers to the study, and so on. Participants were compensated $100 for completing study activities, and an additional $25 for each eligible peer referral enrolled into the study. Recruitment continued until 201 trans women were enrolled.

Measures included self-reported information and HIV antibody testing. An interviewer-administered face-to-face questionnaire gathered information on demographic characteristics, risk behaviors (e.g. injection drug use, exchange sex, partner risks), structural factors (e.g. homelessness, living situation, history of detention), social support (e.g. familial support), health insurance status, use of medical services, and engagement in HIV care. The family support measure was based on a single question, phrased as “I get the emotional help and support I need from my family.” The responses were a Likert scale from strongly agree to strongly disagree. Injection drug use refers to illicit drugs only and does not include prescribed drugs. Participants were asked “Have you ever in your life shot up or injected any drugs other than those prescribed for you? By shooting up, I mean anytime you might have used a needle to inject drugs in your veins, under the skin, or in the muscle.” Venous blood specimens were tested for antibodies to HIV (Chembio Sure Check 1/2 Assay test, Chembio Diagnostics Inc, Hauppauge, NY, USA) and those testing reactive were confirmed with an oral antibody test (Oraquick1 HIV Rapid Antibody Test, OraSure Technologies, Bethlehem, PA, USA). HIV viral load was self-reported.

### Analysis

Using STATA version 17.0 (College Station, TX), we provide univariate analysis of key measures including counts, proportions, and medians with interquartile ranges. Bivariate associations of the above key measures with HIV seropositivity were assessed in cross tabulations using the Hhi square test. Candidate variables for inclusion in a multivariable model were those with *p* ≤ 0.201. We then conducted a stepwise process removing any variables where *p* > 0.05. The final model retained only variables that were significantly associated with HIV at *p* < 0.05. Analyses are unweighted (i.e. show the sample estimates) following the CDC report for the National HIV Behavioral Surveillance among Transgender Women^18^ and noted challenges of RDS weighting and evidence that regression models perform better when unweighted.^21,22^

### Ethical considerations

The protocol was reviewed and approved by the Institutional Review Board of University of California, San Francisco (UCSF) (protocol #15-17775). All participants provided verbal informed consent to preserve anonymity.

## Results

### Participant characteristics and HIV risk-related behavior

The study recruited 201 trans women, with 39.3% age 50 years and older and 3.0% between the ages of 18 and 24 (Table 1).

**Table 1.** Socio-demographic characteristics, familial support, health care access, and HIV-related risk behaviors among trans women, San Francisco, 7/2019–2/2020.

Characteristics	*n*	%^a^
Total	201	100
Age at interview in years		
18–24	6	3.0
25–34	43	21.4
35–39	21	10.4
40–49	52	25.9
50+	79	39.3
Gender identity		
Woman	23	11.4
Trans woman	128	63.7
Woman and trans woman	19	9.5
Other gender identity	31	15.4
Race/ethnicity		
Black/African American, non-hispanic/Latina/e/x	42	20.9
Asian, non-Hispanic/Latina/e/x	15	7.5
Hispanic/Latina/e/x	75	37.3
White, non-Hispanic/Latina/e/x	36	17.9
Other/multiple	32	15.9
Current living situation		
Own/rent	79	39.3
Live with someone without paying rent	13	6.5
Hotel/SRO	68	33.8
Homeless or shelter	41	20.4
Homeless during past 12 months		
No	81	40.3
Yes	120	59.7
Below 2019 HUD^18^ extremely low income		
No	30	14.9
Yes	170	84.6
Education level		
Less than high school	44	21.9
High school/GED	62	30.8
Some college	69	34.3
Bachelor’s degree or higher	25	12.4
Ever held in detention for more than 24 h		
No	66	32.8
Yes	135	67.2
Receives emotional support from family		
Disagree/strongly disagree	82	40.8
Neither agree nor disagree	22	10.9
Agree/strongly agree	97	48.3
Currently on hormones		
Yes	153	76.1
No	37	18.4
Other	11	5.5
Currently insured		
No	15	7.5
Yes	186	92.5
Type of insurance (multiple answers allowed)		
Private health insurance	23	11.4
Medicaid	151	75.1
Medicare	33	16.4
Other government insurance	7	3.5
Veterans Administration	3	1.5
Local public insurance	19	9.5
Lacked health care due to cost, last 12 months		
No	161	80.1
Yes	40	19.9
Injection drug use, ever		
No	139	69.2
Yes	62	30.8
Injection drug use, last 12 months		
No	173	86.1
Yes	28	13.9
Received drugs or money in exchange for sex, last 12 months		
No	128	63.7
Yes	73	36.3
Tested for STD, last 12 months		
No	79	39.3
Yes	117	58.2
Sexual partner who was MSM (lifetime)		
No	70	34.8
Yes	124	61.7
Don’t know	7	3.5
Sexual partner who had other trans women partners (lifetime)		
No	34	16.9
Yes	158	78.6
Don’t know	9	4.5
Sexual partner who injected drugs (lifetime)		
No	75	37.3
Yes	115	57.2
Don’t know	11	5.5
Sexual partner who had been incarcerated (lifetime)		
No	45	22.4
Yes	152	75.6
Don’t know	4	2.0
Median sex partners, last 12 months (IQR)	3	1–6
Median exchange sex partners, last 12 months (IQR)	0	0–2.5

^a^Categories may not add up to total due to missing data or declined to answer.

Most were women of color, with 37.3% Hispanic/Latina, 20.9% Black/African American, 15.9% other or multiple race/ethnicities, 7.5% Asian, and 17.9% White. A majority (59.7%) reported being homeless during the last 12 months; 84.6% earned below the 2019 Housing and Urban Development (HUD) level^18^ of extremely low income. Nearly half (48.3%) of trans women agreed or strongly agreed that they received emotional support from their family. Two-thirds (67.2%) reported incarceration for over 24h in their lifetime. Most (92.5%) had health insurance, with many having Medicaid (75.1%). One in five (19.9%) said they were unable to get health care in the last year due to the cost.

Table 1 also includes indicators of risk for HIV. Lifetime injection drug use was reported by 30.8%, with 13.9% injecting in the last 12 months. Over one-third (36.3%) reported receiving drugs or money in exchange for sex in the last year. Many trans women (61.7%) said they had a sexual partner who was a cisgender man who had sex with cisgender men (i.e. were MSM) in their lifetime, and 78.6% had a sexual partner who had other trans women partners in their lifetime. A majority (57.2%) of trans women reported having a sexual partner who had ever injected drugs; 75.6% had a sexual partner who had been previously incarcerated.

### Correlates of HIV infection

HIV prevalence was 42.3% (Table 2).

**Table 2.** HIV prevalence by socio-demographic characteristics, familial support, health care access, and HIV-related risk behaviors among trans women, San Francisco, 7/2019–2/2020.

Characteristics	HIV negative *n*	%	HIV positive *n*	%	*p*-value*
Total	116	57.7	85	42.3	
Age at interview (years)					0.036
18–24	6	100	0	0.0	
25–34	30	69.8	13	30.2	
35–39	11	52.4	10	47.6	
40–49	31	59.6	21	40.4	
50+	38	48.1	41	51.9	
Gender identity					0.861
Woman	14	60.9	9	39.1	
Trans woman	71	55.5	57	44.5	
Woman and trans woman	12	63.2	7	36.8	
Other gender identity	19	61.3	12	38.7	
Race/ethnicity					0.004
Black/African American, non-hispanic/Latina/e/x	14	33.3	28	66.7	
Asian, non-Hispanic/Latina/e/x	9	60.0	6	40.0	
Hispanic/Latina/e/x	49	65.3	26	34.7	
White, non-Hispanic/Latina/e/x	26	72.2	10	27.8	
Other/multiple	19	59.4	14	43.8	
Current living situation					0.236
Own/rent	40	50.6	39	49.4	
Live with someone without paying rent	6	46.2	7	53.8	
Hotel/SRO	44	64.7	24	35.3
Homeless or shelter	26	63.4	15	36.6	
Homeless during past 12mon					0.275	
No	43	53.1	38	46.9	
Yes	73	60.8	47	39.2	
Below 2019 HUD extremely low income					0.92
No	17	56.7	13	43.3	
Yes	98	57.6	72	42.4
Education level					0.100
Less than high school	24	54.5	20	45.5	
High school/GED	32	51.6	30	48.4	
Some college	39	56.5	30	43.5	
Bachelor’s degree or higher	20	80.0	5	20.0	
Ever held in detention for more than 24 h					0.003	
No	48	72.7	18	27.3	
Yes	68	50.4	67	49.6	
Receives emotional support from family					0.003
Disagree/strongly disagree	59	72.0	23	28.0	
Neither agree nor disagree	11	50.0	11	50.0	
Agree/strongly agree	46	47.4	51	52.6	
Currently on hormones					0.542
Yes	85	55.6	68	44.4	
No	24	64.9	13	35.1	
Other	7	63.6	4	36.4	
Currently insured					0.018
No	13	86.7	2	13.3	
Yes	103	55.4	83	44.6	
Private health insurance					0.745
No	102	57.3	76	42.7	
Yes	14	60.9	9	39.1	
Medicaid					0.003
No	38	76.0	12	24.0	
Yes	78	51.7	73	48.3	
Medicare					0.052
No	102	60.7	66	39.3	
Yes	14	42.4	19	57.6	
Other government insurance					0.975
No	112	57.7	82	42.3	
Yes	4	57.1	3	42.9	
Veterans Administration coverage					0.752
No	114	57.6	84	42.4	
Yes	2	66.7	1	0	
Local public insurance					0.638
No	106	58.2	76	41.8	
Yes	10	52.6	9	0	
Lacked health care due to cost, last 12 months					0.005
No	85	52.8	76	47.2	
Yes	31	77.5	9	22.5	
Injection drug use, ever					<0.001
No	92	66.2	47	33.8	
Yes	24	38.7	38	61.3	
Injection drug use, last 12 months					0.011
No	106	61.3	67	38.7	
Yes	10	35.7	18	64.3	
Received drugs or money in exchange for sex, last 12 months					0.394
No	71	55.5	57	44.5	
Yes	45	61.6	28	38.4	
Tested for STD, last 12 months					0.365
No	42	53.2	37	46.8	
Yes	72	61.5	45	38.5	
Sexual partner who was MSM (lifetime)					0.373
No	43	61.4	27	38.6	
Yes	68	54.8	56	45.2	
Sexual partner who had other trans woman partners (lifetime)					0.201
No	23	67.6	11	32.4	
Yes	88	55.7	70	44.3	
Sexual partner who injected drugs (lifetime)					<0.001
No	57	76.0	18	24.0	
Yes	50	43.5	65	56.5	
Sexual partner who had been incarcerated (lifetime)					0.011
No	33	73.3	12	26.7	
Yes	79	52.0	73	48.0

Missing, don’t know, and refused responses were removed from analyses. *Chi-square test

HIV prevalence increased with increasing age, with no infections detected among trans women age 18–24 years, while HIV prevalence was 51.9% among women age 50 and older. Black/African American trans women had the highest prevalence of HIV (66.7%) among race/ethnicities while Whites had the lowest (27.8%). Being held in detention for more than 24h was associated with higher prevalence of HIV (49.6% vs. 27.3%, *p* = 0.003). HIV prevalence was higher among women who agree/strongly agreed with the statement that they received emotional support from their family than those who disagreed (52.6% vs. 28.0%, *p* = 0.003). Having health insurance was associated with higher HIV prevalence (44.6% vs 13.3%, *p* = 0.018), as was having Medicaid as the type of health insurance (48.3% vs 24.0%, *p* = 0.003). Additionally, HIV prevalence was higher among trans women who said they had trouble accessing health care due to cost compared to those who did not. HIV prevalence was higher among trans women who reported a history of injecting drugs ever (61.3%) and in the last 12 months (64.3%). Having a sexual partner with a history of injecting drugs was also associated with higher HIV prevalence (56.5%), as was having a partner who had a history of incarceration (48.0%).

Independent correlates of HIV infection in multivariable analysis are shown in Table 3.

**Table 3.** Multivariable logistic regression analysis results, independent associations with HIV prevalence, trans women in San Francisco, 7/2019–2/2020.

Associated factor	Adjusted odds ratio	95% confidence interval	*p*-value
Sexual partner who injected drugs (lifetime)	3.30	1.58, 6.90	0.002
Injection drug use, ever	2.28	1.06, 4.89	0.035
Did not lack care due to cost, last 12 months	2.63	1.02, 6.67	0.045
Receives emotional support from family	2.85	1.43, 5.65	0.003
Black/African American, non-hispanic/Latina/e/x	2.59	1.16, 5.79	0.021

The correlate of HIV infection with the strongest magnitude of association was having a partner who had ever injected drugs (adjusted odds ratio [AOR] 3.30, 95% confidence interval [CI] 1.58–6.90). The respondent’s own injection drug use (AOR 2.28, 95% 1.06–4.89) was also significantly associated with HIV infection. Cost not being a barrier to health care continued to be associated with HIV infection (AOR 2.63, 95% CI 1.02, 6.67). Having emotional support from family was associated with having HIV (AOR 2.85, 95% CI 1.43, 5.65). Lastly, HIV infection remained significantly higher among Black/African American women (AOR 2.59, 95% CI 1.16, 5.79) compared to other groups.

### HIV care cascade

**Figure 1.**
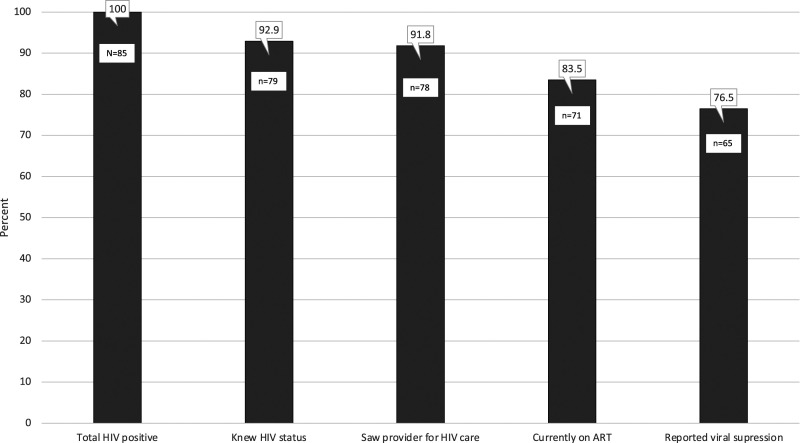
Engagement of trans women in HIV care, San Francisco, 2020.

Of 85 trans women testing HIV positive in our sample, 79 (92.9%) reported having a previous positive test result (i.e. were aware of their HIV infection), 78 (91.8%) had seen a provider for HIV care, 71 (83.5%) were currently on ART, and 65 (76.5%) reported that they were virally suppressed on their most recent viral load (Figure 1).

Using the 90–90–90 framework, 92.9% of trans women with HIV were previously diagnosed, of whom 89.9% (71/79) were on ART, of whom 91.5% (65/71) reported being virally suppressed.

## Discussion

Our survey found evidence of progress in the response to HIV among trans women in San Francisco as well as persistent challenges through 2020. Our data indicate that the 90–90–90 targets for engagement in HIV care have been met for trans women in San Francisco, at 93–90–92. This follows a history of trans women in San Francisco falling behind other populations at risk for HIV. As noted above, estimates for 2017 showed that trans women fell short on being in care/ART uptake (79%) and viral suppression (82%).^11^ Ten years ago, being on ART and being virally suppressed were substantially lower at 68% for each.^3^ However, HIV prevalence among trans women remains high, measured at 32.1% in 2017^5^ and now at 42.3% in 2020.

Our data are included in the CDC’s first surveys of trans women in multiple cities in the United States as part of the NHBS.^18^ HIV prevalence was 42% across the seven cities surveyed, which included Atlanta, Los Angeles, New Orleans, New York City, Philadelphia, and Seattle, in addition to San Francisco. Highest prevalence of HIV was found among trans women who identified as American Indian/Alaska Native (65%) and Black/African American (62%). PrEP awareness was high (92%), while use was low (32%) with several barriers identified including medical mistrust and lack of trans-inclusive marketing.^18^ The report found accessing gender affirming medical treatment to be positively associated with HIV medical care.^18^

Our study may shed light on the source of such high prevalence of HIV among trans women. An epidemiological conundrum has been why trans women experience the highest prevalence of HIV when their reported sexual partners, men attracted to women, have the lowest in San Francisco.^15,20,23^ We found the single strongest association with HIV positivity was having a partner with a history of injection drug use. This association was independent of their own history of injection drug use, which also remained strongly predictive of HIV infection in multivariable analysis. Previous analysis of phylogenetic clusters that included the strains from trans women suggested a similar pattern – that sexual networks that include men who inject drugs may be the avenue for high transmission of HIV to trans women.^14^ In addition, programmatic data from HIV testing programs found that the partners of trans women were significantly more likely to have used and injected drugs in the past 12 months than other testers.^16^ In contrast, having sexual partners who are also MSM and having sexual partners who have other trans women partners, while commonly reported, were not associated with HIV infection in the current data. Moreover, engaging in commercial sex work was also not associated with HIV infection. Unfortunately, direct data from the partners of trans women remain scarce. Future research that includes the difficult-to-reach population of partners will be needed to confirm the epidemiology of HIV among trans women. Meanwhile, harm reduction and HIV prevention programs need to reach the partners of trans women, possibly through trans women themselves. At the same time, involving trans women to work with their partners might be difficult and put them at risk for violence. Such interventions will need to concurrently address interpersonal violence. Of note, PrEP uptake among people who inject drugs is remarkably low in San Francisco given the high levels achieved among MSM and more recently among trans women.^24^ Uptake in PrEP among people who inject drugs may have substantial impact on HIV transmission to trans women.

Our survey confirmed that the significantly higher prevalence of HIV among Black/African American women noted in prior studies in San Francisco^5,25^ persists. Two-thirds of Black/African-American trans women in our study (66.7%) were living with HIV in 2020. The figure for San Francisco is similar to the overall 62% prevalence of HIV among Black/African American trans women in the seven cities included in the CDC’s NHBS.^5,18^ Previous research has suggested causes of this disparity may be increased barriers to prevention and care services due to intersectional stigmatization by transphobia and racism and the sequelae such as incarceration, few employment opportunities, discrimination,^5^ and unstable housing.^6,26^ Addressing root causes such as systemic racism and intersecting stigmas will be challenging. Meanwhile, HIV prevention and care resources need to prioritize Black/African American trans women.

On a positive note, our study found that social, emotional, and medical support were associated with HIV positive status. High medical and social support among trans women living with HIV speaks to the strength of the HIV care and support system in San Francisco. Trans women living with HIV, especially those who are older, may have had the time and support networks to develop chosen families, which may explain higher familial support. Familial support has been associated with better mental health, higher levels of condom use, and less unprotected sex among trans women.^27,28^ Therefore, interventions may be needed to increase familial support, including development of chosen family support, among trans women not living with HIV.

We acknowledge limitations of our data. First, key outcomes along the care cascade are based on self-reported data, such as prior test results, ART use, and most recent viral load. Self-report may be overly optimistic and overestimate the amount of virally suppressed trans women and trans women on ART in San Francisco. Direct measures of viral load and antiretroviral metabolites would provide more precise data. While our interviewers were trans women and specifically trained on the instrument, there is the possibility of social desirability bias when answering sensitive questions. Second, our survey may not be fully representative of the population. It has been previously suggested that RDS survey methods may reach only the lower socio-economic strata of trans women,^29^ possibly due to the monetary incentive for participation. Even within the lower socio-economic status groups, some segments of the population may not have participated. For example, two-thirds of trans women in our sample had a history of incarceration, with one in seven incarcerated in the last year. We also note that our sample had a high proportion of women over 50 and may not represent young trans women. Our study was not able to sample trans women incarcerated at the time of the survey. External validity may be limited as trans women in San Francisco may not be representative of other cities in the US. Nonetheless, a strength of our sample was its high diversity with respect to race/ethnicity. Lastly, the cross-sectional design limits inference on causality, or the direction of effect and cause may be reversed. For example, as noted above, having greater medical support is likely an effect of an HIV diagnosis with subsequent eligibility for health insurance, not the cause.

Our data show HIV prevalence remains high and rising, Black/African American trans women remain disproportionately affected, and indicators of sexual and drug use risks also remain high. The rising prevalence of HIV is likely due in part to increased survival following improvement in ART use and viral suppression. Our study points to the positive news that the 90–90–90 targets for trans women were met as of the present study’s data collection in 2019–2020. Nonetheless, HIV transmission continues, with moderate to high rates measured as recently as 2020.^30^ Our data also point to areas for prioritization to strengthen the HIV epidemic response for trans women. The strong association with injection drug use and partner injection drug use point to a need for better data on the partnerships of trans women and ways to reduce risk of injection for trans women. Of note, there has been improvement of PrEP awareness and uptake among trans women in San Francisco. In 2018, 79% of HIV negative trans women were aware of PrEP, 35% had talked with a provider about PrEP, and 15% had used PrEP in the last 6 months.^4^ With targeted programs, a year later 94% of HIV negative trans women were aware of PrEP, 65% had talked with a provider about PrEP, and 45% had used PrEP in the last 12 months.^7^ A gap in HIV prevention is the partners of trans women. Interventions are needed that reach the partners of trans women through referrals, ensure retention on ART if HIV positive, and offering of PrEP if HIV negative.
